# On Some Unrecognised Forms of Mental Disorder

**Published:** 1856-04-01

**Authors:** Forbes Winslow


### ART. VII.—ON SOME UNRECOGNISED FORMS OF MENTAL DISORDER.

BY FORBES WINSLOW, M.D.

(*Continued from page* 90.)

I NOW proceed to cite a few illustrations of this *type* of undetected insanity. A lady, who up to the age of forty-three was never known to manifest anything resembling a passionate disposition or a bad temper, became, after the birth of her last child, subject to paroxysms of overpowering and ungovernable passion, induced by the most trifling and apparently insignificant causes. This continued for several years, her state of mind never being considered otherwise than sound. I had several opportunities, after her morbid condition was recognised, of observing her fits of rage; and certainly I never witnessed any demonstrations off the stage so truly appalling. Her intellect was not deranged, *id est*, there was no *aberration of idea* in connexion with the case; there was no appreciable delusion, no perversion of the affections, and no hallucinations of the senses. Her mental affection manifested itself solely in these sudden paroxysms of intense passion. These attacks generally occurred once a week, sometimes only once during the month; but for a short period she had them more frequently. They were almost invariably preceded by vertigo, pain in the occipital region, and a dimness of vision. It was the presence of these physical symptoms that led to the supposition of the existence in this case of some undetected cerebral mischief. I ordered leeches to the head, a few days in advance of the expected paroxysms; regulated the bowels and secretions, and thus greatly diminished the intensity of the passionate excitement, but failed in entirely curing the case. Dr. Cheyne refers to a somewhat similar instance in his work on "Partial Derangement of Mind

ISSUE

associated with Religious Impressions." He says, a friend of his was one day riding with a clergyman of refined manners, who for many years had been devoted to the service of God. To the amazement of his friend, his companion, without any adequate provocation, fell into a paroxysm of ungovernable fury, swearing at a wood-ranger, and threatening him with vengeance, because he had been dilatory in obeying an order which he had received relative to a matter of little importance. Had (observes Dr. Cheyne) this fact become public, all the devotedness to his profession, for which this excellent man was distinguished, would by many have been considered as assumed, and his habitual humility of demeanour, arising from a sense of his own unworthiness, as the result of hypocrisy. It appears that this gentleman had a short time previously undertaken a duty which led to over-excitement of the brain. He was quite conscious of the incongruity of his conduct. It appears that his only brother had died in an asylum. I have a young lady under treatment whose only appreciable morbid condition is that of being subject to violent and uncontrollable fits of passion. These attacks frequently occur during the night. The poor little creature is painfully conscious of her sad infirmity, and assures me that she struggles heroically against it. We sometimes in practice see a modified form of this affection exhibiting itself in a bad, morose, and capricious disposition, called by Dr. Marshall Hall, who has seen several of these cases, "*temper disease*." This affection is not, however, confined to the female sex. A celebrated member of the House of Commons, now dead, had periodical attacks of this nature, particularly after his brain had been overwrought. I was informed by a particular friend of the gentleman to whom I refer, that he once saw him in a terrible paroxysm of fury after making an election speech. He was perfectly conscious that at these periods he was temporarily beside himself. He was in the habit of dashing cold water over his head during the fit, and occasionally when suffering from much physical exhaustion he has been known, with great benefit, to drink at a draught a pint of port wine. The celebrated Spanish General Galvez was subject to attacks of this nature. A bottle of claret was his remedy. It immediately composed his mind, probably, as Dr. Rush remarks, by overcoming a weak, morbid action, and producing agreeable and healthy excitement of brain. Would not, adds Dr. Rush, a dose of laudanum have been a better remedy for the purpose? A young gentleman was thrown from his horse, and fell upon his head. For ten minutes after the accident he continued in a state of coma. Since his recovery he has been subject to fits of passionate excitement. These attacks are generally preceded by severe headaches. His mental faculties do not appear much, if at all, impaired, but he continues to suffer from these morbidly painful ebullitions of temper. Prior to the injury, he exhibited the most extraordinary degree of self-control and equanimity of temper. Dr. Beddoes refers to the case of a lady, who, after her recovery from an attack of brain fever, became extremely irascible. This was the reverse of her natural disposition. She made herself so offensively disagreeable to all her family, that her husband, a most amiable and self-denying man, was compelled to separate himself from her, and abandon his once happy fireside.

A somewhat similar case I visited in consultation with Dr. Webster. In this instance the lady was in the habit, during her paroxysms of passion, of seizing hold of her husband's hair and tearing it out by handfuls. This poor fellow has often come to me in great distress, having a full assurance of his wife's insanity, beseeching me to protect him from her acts of insane violence. She was clearly disordered in her mind, but neither Dr. Webster nor myself could detect, in our consultations with her, sufficiently conclusive evidence to justify us in signing a medical certificate authorizing her confinement. We both lamented that, owing to a defective state of the law, we could not grapple with the case; but in this, as in numerous anomalous instances of disordered mind,we felt that nothing could be done, and matters must be allowed to take their course.

I have referred to a certain morbid mental condition, exhibiting itself exclusively in acts of cruelty and brutality. This form of unrecognised disorder may exist unassociated with delusion. There is much of this latent and undetected alienation of mind in real life, producing, within the sacred precincts of domestic life, great irregularities of conduct and a fearful amount of domestic misery. It often coexists with great talents and high attainments, and is compatible with the exercise of active philanthropy and benevolence. The ordinary actions or conversation of those so affected, in many cases, would not convey to a stranger an idea of the existence of such a sad state of the intellect. Howard, the celebrated philanthropist, affords an unhappy illustration of this type of disorder. He is represented to have been a tyrant in his own house. His cruel treatment caused the death of his wife. He was in the habit, for many years after her death, of doing penance before her picture. He had an only son, whom, for the slightest offence, he punished with terrible severity. He was in the habit of making this son stand for hours in a prescribed grotto in the garden. The son became a lunatic, as the result of this brutal treatment. Several similar cases have been brought under my observation. In one instance, temporary confinement was resorted to, but without positive advantage.

ISSUE

The paroxysms of ungovernable brutality returned immediately after the patient's return home.

A lady, moving in good society, happily married, accomplished, well educated, of sweet temper, and with a mind under the controlling influence of religious principles, manifested, at the age of forty-five, an extraordinary change of character and habits. She became irritable from trifling causes; was continually quarrelling with her husband and servants; discharged her tradesmen, accusing them of acts of dishonesty; and offended many of her most intimate friends and relations by her cold, and often repulsive manner. This state of mind continued for two years, during which period she played the capricious tyrant within the sphere of the domestic circle. Her husband became nearly broken-hearted; his friends and relations could not enter his house without being insulted; he neglected his business, and his health became seriously impaired from constant anxiety. A new phase of the malady, however, exhibited itself. She one day accused her husband of gross infidelity. Proof was demanded. She immediately produced several anonymous letters which she had received, containing a minute, circumstantial, and apparently truthful account of her husband's misconduct. These letters appeared to substantiate, as conclusively as such documentary evidence could do, the accusations. No person doubted the genuineness of these letters. Her friends, however, refused to recognise, even at this time, her actual morbid state of mind. She subsequently had an epileptic seizure, followed by partial paralysis. I then saw the case. Her cerebral condition being then apparent, she was removed from home. It was now discovered, beyond a doubt, *that this lady had written the anonymous letters to herself, accusing her husband of infidelity,—had addressed* and posted them, *and had eventually become impressed with the conviction that the letters were actually written by a stranger*, *and contained a true statement of facts.* They had, as it afterwards appeared, been concealed about her person for nearly six months!

Not many months back, I was requested to visit a lady, who, after a painful and dangerous accouchement, exhibited, without any adequate exciting cause, an inveterate feeling of hatred towards one of her children. She treated this child with great and systematic brutality; and to such an extent did she carry this morbid and unnatural feeling, that her husband was obliged to remove the child from the house, and to place it under the care of a relative in a distant part of the country. I had no doubt at the time that this person's mind was disordered. Such was my written opinion. The idea was, however, repudiated by nearly all the members of the family, who obstinately closed their eyes to her sad and melancholy condition. The only evidence that existed, at that period, of mental disorder, was her unnatural alienation of affection, and her brutal conduct towards one of her children. This state of mind appeared unassociated with any appreciable delusive ideas. Three weeks had scarcely elapsed since my first consultation in this case, when I was informed that this lady had made an unsuccessful attempt at suicide. It was then obvious that she was not in a sane state of mind, and her family no longer hesitated in placing her in a private family, under close restraint. We occasionally observe evidences of this morbid state at a very early period of life, and it is indicative of an original organic defect in the constitution of the intellect.

I cite the following case from the *Times*:—Thomas Pepper, fourteen years of age, a pot-boy, a clever lad, but of sullen and morose disposition, committed suicide by hanging himself in an arbour in his master's bowling-green. It appeared from the evidence that the mind of the deceased was peculiarly formed, his conduct frequently evincing a predisposition to cruelty. He had been frequently known to hang up mice and other animals for the purpose of enjoying the pain which they appeared to suffer whilst in the agonies of death. He would often call boys to witness these sports, exclaiming—"Here's a lark; he is just having his last kick." He had often been known to catch flies and throw them into the fire, that he might observe them whilst burning. He had also been observed, whilst passing along the street, to pull the ears of the children—lifting them off the ground by their ears; and when they cried out with pain, he would burst out into a fiendish paroxysm of delight at their sufferings. Witnesses deposed that about four years previously, when only ten years of age, he attempted to strangle himself, in consequence of his mother having chastised him. He locked himself up in a room, and, when discovered, life was nearly extinct. I refer to this as an illustration of a *type* of mental disorder arising from a congenital mal-organization of the brain and intellect. This morbid disposition may be either connate, hereditary, or may be the *sequelæ* of disease affecting the healthy condition of the brain. It occasionally supervenes upon injuries of the head.

Mr. Shute, surgeon, of Mecklenburgh-square, consulted me respecting a youth whose whole moral character had become completely changed in consequence of a severe injury that he had sustained. This young gentleman, when of the age of eighteen or nineteen, was attacked by fever. In a paroxysm of delirium he sprung violently out of bed, and severely cut his ankle; considerable hæmorrhage followed. After his recovery, his whole moral character was found to have undergone a complete metamorphosis. From being a well-conditioned boy, kind and affectionate to his parents, steady in his habits, sober, of unimpeachable veracity,—he became a drunkard, a liar, a thief, and lost all sense of decency and decorum! His intellectual faculties were unaffected. He was clever, intelligent, sharp-witted, but his every action was perfectly brutal. This boy, prior to his illness, was known to hang with endearing affection round the neck of his mother; but after this sad change, I have seen him attack her with brutal and savage ferocity. This patient was for some years in close confinement. He was subsequently sent abroad; but during a voyage to the East Indies he mysteriously disappeared one evening from the quarter-deck of the ship, and is supposed to have committed suicide by jumping into the sea. We occasionally meet another *type* of unrecognised mental disorder. I refer to cases in which there appears to be a *paralysis of the moral sense.* Such cases are not inappropriately termed moral idiots.

A young gentleman, who had been greatly indulged and petted at home, exhibited, shortly after going to school, a morose, cruel, and revengeful disposition. He quarrelled with the other boys—committed several petty acts of robbery, accusing others of being the culprits. He pursued his studies with intelligence, and was generally at the head of his class. His conduct became so systematically brutal, savage, and untruthful, that his father was requested peremptorily to remove him. The gentleman under whose care the youth was placed, was induced, by the earnest persuasions of the father, to withdraw his request and retain the boy. For several days he was noticed to be unusually taciturn. He was perceived to be busily occupied one morning in writing: being called suddenly out of the room, his letter was examined, and it was found to contain the details of a plan he had carefully concocted for the murder of one of the other boys, towards whom he entertained feelings of rancorous animosity. His letter was written to a boy who had left the school for misconduct, and who appeared to be his confidant. He had procured a long, sharp-pointed bodkin, which he intended, whilst his victim was asleep, to drive into his heart by means of a hammer which he had in his possession. In the letter, giving a minute description of the contemplated murder, he says—"*To-night I will do for the little devil.*" This boy was immediately placed under the care of his father, and at the advice of an eminent provincial physician, he was, without loss of time, subjected to close restraint. I am informed, that there is now no doubt of his insanity. I did not see this case myself, but I obtained these particulars from the father of the young gentleman who had so narrow an escape of his life. If this youth had committed murder, what would have been the verdict of the jury?

N. B., ætat. sixteen, of singularly unruly and intractable character, selfish, wayward, violent without ground or motive, and liable, under paroxysms of his moodiness, to do personal mischief to others. He was not, however, of a physically bold character. He was of fair understanding, and exhibited considerable acuteness in sophistical apologies for his wayward conduct. He made little or no progress in any kind of study. His fancy was vivid, supplying him profusely with sarcastic imagery. He was subjected at different times to a firmly mild and to a rigid discipline. Solitary confinement was tried, but to this he was impassive. He was sent to school, where he drew a knife upon one of the officers of the establishment, and produced a deep feeling of aversion in the minds of his companions by the undisguised pleasure which he showed at some bloodshed which took place in the town during a political disturbance. He manifested no sensual disposition, and was careful of property. His conduct became worse, and more savagely violent to his relatives. It is recorded that, at the early age of thirteen, he stripped himself naked and exposed his person to his sisters. I am indebted to Dr. Mayo for this interesting illustration of what I term moral idiocy, or congenital depravity. When referring to this painfully anomalous class of affections, the late Dr. Woodward, Physician to the State Lunatic Assylum of Massachusetts, observes,—

"Besides a disease of the moral powers there seems to me to be in some cases something like moral idiocy, or such an imbecile state of the moral faculties from birth as to make the individual irresponsible for his moral actions. The persons to whom I refer have rarely much vigour of mind, although they are by no means idiots in understanding."

A boy under Dr. Haslam's care, only thirteen years of age, appeared to possess no one of the moral faculties, and yet he was conscious of his lamentable state; he often asked, "why God had not made him like other men." Has not Shakspeare placed in Edgar's mouth a faithful portrait of this class of case? When delineating his own character, Edgar exclaims,—

"I was a serving man, proud in heart and mind,
That served the lust of my mistress's heart,
And did the act of darkness with her;
Swore as many oaths as I spake words;
Wine I loved deeply, dice dearly:
I was false of heart, light of ears, and bloody of hand;
Hog in filth, fox in stealth, wolf in greediness,
Dog in madness, lion in prey."

A boy, in early life, was struck violently upon the head when at school by a brutal fellow employed as usher. He was partially stunned, but recovered from the effects of the injury. When of sufficiently advanced age, he joined his father in business. He became subject to attacks of headache, particularly if exposed to much anxiety. For some months he continued sullen, was often absent from the counting-house, became the associate of the lowest class of society, and was detected in abstracting several large sums of money from his father's private desk. In this condition he remained for seven or eight months, no one suspecting the morbid state of his intellect. One morning whilst sitting in the counting-house, he suddenly seized one of the clerks by the throat and attempted to throttle him. A severe scuffle ensued. Upon separating the combatants, it was discovered that the gentleman's mind was obviously affected. He became suddenly, as it were, demoniacally possessed. He poured fourth a volley of filthy oaths, and an amount of obscenity appalling to those about him. There appeared no impairment of the reasoning powers, of the memory, or reflective faculties. He suddenly lost all perception of truth, decency, and propriety. I saw this poor fellow in several of his paroxysms, and must confess, if I were disposed to believe in the possibility of demoniacal possession, I should cite this case as one illustrative of the fact. I have referred to instances of unrecognised monomania floating upon the surface of society. I am acquainted with two existing cases of this form of mental disorder where disease of the mind is not suspected. The affection exhibits itself in unreasonable and morbid hatred to one member of the family. One child, without any valid reason, is ostracised by his mother from home, in consequence of her morbid hatred of him. In the other case one out of a large family is treated with great harshness, and occasionally with cold neglect, by his only parent. In both these instances, I have no doubt some monomaniacal idea exists, but is unrecognised. Having been consulted in one of these cases, I have recommended the son to whom, unhappily, the concealed delusion relates, to issue a Commission of Lunacy, with the view of protecting his pecuniary interests. On all other points, no mental infirmity can be detected; and with regard to her other children, her affections remain intact. I feel quite assured that this lady has no ground for this unnatural feeling. These latent and unrecognised attacks of monomania frequently lead to overt acts of violence, crime, brutality, and suicide, and very often to alienation of property, no departure from healthy mind being suspected. A few years back, I received a summons from Mr. Gilbert Abbott A'Beckett, the police magistrate, to examine a case of alleged insanity. It appears that a labouring man had committed several serious assaults, and had consequently been arrested by the police, and temporarily confined. This man was examined by a medical gentleman, who pronounced him to be a lunatic, without being able to assign sufficient specific reasons for such an opinion. Mr. A'Beckett had, on more than one occasion, investigated the case, and had taken the evidence of the medical man referred to, but could detect no insanity in the man's appearance or conversation. The medical gentleman asserted it to be his belief that the prisoner was insane, but could give no satisfactory reason for this opinion beyond the man's apparently unreasonable conduct and mad acts of violence. I obeyed Mr. A'Beckett's summons, and had to examine the prisoner publicly in court. It was not until after the expiration of nearly half-an-hour that I obtained any semblance of a clue to the actual state of the man's mind. I subsequently discovered that he was unequivocally a monomaniac. He believed that a strange person, having evil designs upon him, was in the habit of placing daily a small pill upon the mantelpiece of his bed-room; that this pill (which he was compelled to swallow) contained an ingredient that greatly excited him, destroyed all power of self-control, and led him to commit the acts of violence of which he stood charged. His insanity then became obvious, and Mr. A'Beckett, without any hesitation, signed a warrant committing him to an asylum. It appears that this poor fellow had been severely punished on previous occasions for different acts of violence, no one suspecting the existence of mental disorder. It was not until I had subjected him to a close and rigid examination for nearly three-quarters of an hour, during which the lunatic showed extraordinary ingenuity in parrying my questions, that I could establish, with satisfaction to myself, the presence of this monomaniacal idea. Let us charitably hope, that many extraordinary and apparently unreasonable and motiveless acts of brutality, violence, cruelty, passion, and crime, that appear to result from trifling and inadequate exciting causes influencing naturally weak, badly-organized minds, may have their origin in some latent and concealed insanity, and arise from a morbidly uncontrollable and unrecognised mental delusion. Is not the sad history of crime fraught with illustrations of this kind?

(*To be continued.*)

